# Self-expanding metal stent procedure for afferent loop syndrome with ascending cholangitis caused by remnant gastric cancer: A case report

**DOI:** 10.1097/MD.0000000000013072

**Published:** 2018-12-14

**Authors:** Ra Ri Cha, Su Beom Cho, Wan Soo Kim, Jin Joo Kim, Jae Min Lee, Sang Soo Lee, Hyun Jin Kim, Jin Kyu Cho

**Affiliations:** a Department of Internal Medicine, Gyeongsang National University Changwon Hospital, Changwon; b Department of Internal Medicine; c Department of Surgery, Gyeongsang National University Hospital, Gyeongsang National University School of Medicine, Jinju; d Department of Radiology, Gyeongsang National University Changwon Hospital, Changwon, Republic of Korea.

**Keywords:** afferent loop syndrome, Billroth anastomosis, partial gastrectomy, remnant gastric cancer, self-expanding metal stent

## Abstract

**Rationale:**

Self-expanding metal stent placement is a useful procedure for intestinal obstruction. Afferent loop syndrome after gastrectomy is an uncommon complication of gastroenterostomy reconstruction. Ascending cholangitis caused by afferent loop syndrome is a potential, but rare, complication.

**Patient concerns:**

A 73-year-old man with abdominal pain and vomiting was admitted to the emergency room. His medical history was significant for subtotal gastrectomy with Billroth II anastomosis for benign gastric ulcer perforation 40 years prior. He had notable tenderness to palpation, particularly on the epigastric area, and a temperature of 39.0°C.

**Diagnosis:**

Abdominal computed tomography revealed afferent loop syndrome with ascending cholangitis caused by remnant gastric cancer.

**Interventions:**

Percutaneous catheter drainage for management of ascending cholangitis was performed on the day of admission. He was subsequently treated with self-expandable metal stent insertion into the stenotic lesion.

**Outcomes:**

After treatment with percutaneous transhepatic insertion of a self-expanding stent, the patient achieved complete resolution of symptoms. The patient died of disease progression 2 months later, without further recurrence of afferent loop syndrome.

**Lessons:**

Our case shows that insertion of a metal stent via percutaneous transhepatic biliary drainage (PTBD) can effectively treat ascending cholangitis and resolve afferent loop syndrome in inoperable patients.

## 1 Introduction

Afferent loop syndrome is a recognized complication following partial gastrectomy with the Billroth II anastomosis. Traditionally, surgery is considered the best method for the treatment of afferent loop syndrome. Recently, advances in endoscopic and percutaneous techniques have been described in the literature to offer additional options for management of these cases.

Remnant gastric cancer is defined as gastric cancer arising from the remnant stomach at least 5 years after distal gastrectomy for a benign disease.^[1]^ The incidence of remnant gastric cancer has been reported to be 1% to 2%.^[2]^ It is frequently diagnosed at an advanced stage with a low chance of cure, high rate of lymph node metastasis, and a consequent poor prognosis compared to primary gastric cancer.^[3]^ The development of afferent loop syndrome due to remnant gastric cancer is an extremely rare event.

Afferent loop obstruction is usually caused by mechanical obstruction from kinking of the afferent limb, tumor recurrence, adhesions, radiation-induced stenosis, or internal hernias.^[4]^ Obstruction of the afferent loop with progressive accumulation of biliary, pancreatic, and intestinal secretions results in afferent loop dilatation, subsequent dilatation of the biliary tract, cholangitis, and pancreatitis.

Here, we present a case of a 73-year-old male who presented with cholangitis symptoms secondary to afferent loop obstruction due to remnant gastric cancer, who previously underwent partial gastrectomy with Billroth II anastomosis. He was successfully treated with percutaneous transhepatic insertion of a self-expanding stent with favorable outcome.

## 2 Case report

**Figure 1:**
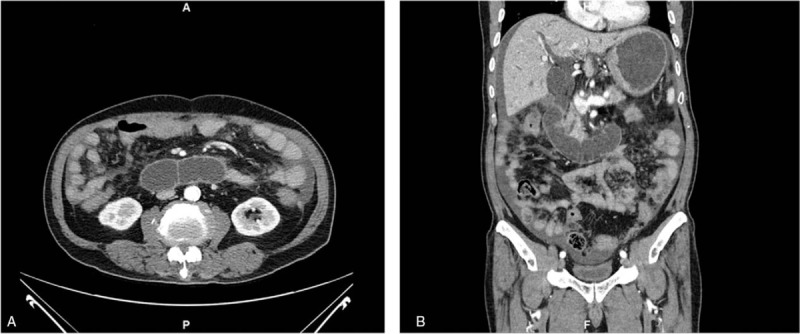
Abdominal contrast-enhanced computed tomography on admission. Images showed afferent loop obstruction (A), intrahepatic bile duct dilatation, and a large volume of ascites (B).

**Figure 2:**
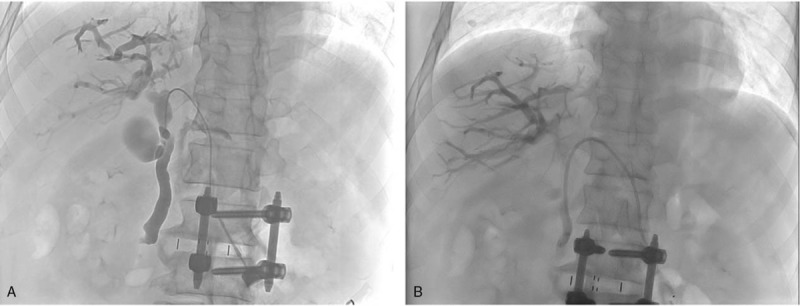
Percutaneous transhepatic biliary drainage for the treatment of malignant afferent loop obstruction. An 8.5 French, multi side hole pigtail catheter tip was inserted via the dilated left intrahepatic duct and, under fluoroscopy, the catheter tip was placed in the common bile duct.

A 73-year old man presented with abdominal pain and vomiting for 1 week. He had a history of early satiety and weight loss of 5 kg in the previous month. His medical history included subtotal gastrectomy with Billroth II anastomosis for benign gastric ulcer perforation 40 years prior. Physical examination showed the following: blood pressure, 120/80 mm Hg; heart rate, 105 beats/minute; respiratory rate, 20 respirations/minute; temperature, 39.0°C. On physical examination of the abdomen, he had marked tenderness, particularly of the epigastric area. The results of laboratory test were as follows: white blood cell count, 10,870 /mm^3^; hemoglobin, 9.0 g/dL; platelet count, 247,000 /mm^3^; albumin, 3.3 g/dL; total/direct bilirubin, 2.06/1.38 mg/dL; alanine aminotransferase, 45 U/l; aspartate aminotransferase, 133 U/l; alkaline phosphatase, 172 U/I; CA 19-9, 165.95 U/Ml. Abdominal computed tomography showed prominent dilatation of the duodenal loop, common bile duct, and bilateral intrahepatic ducts, and a large amount of ascites (Fig. 1). Based on these findings, the patient was diagnosed with afferent loop syndrome complicated by biliary tree dilatation; he was suspected to have ascending cholangitis. As a result of his poor general condition and the presence of ascites suggesting peritoneal seeding, surgical treatment was not an option. Therefore, a sonography-guided PTBD (percutaneous transhepatic biliary drainage) procedure was performed by inserting an 8.5 French, multiside hole pigtail catheter tip via the dilated left intrahepatic duct, with the catheter tip placed in the common bile duct, under fluoroscopy (Fig. 2).

**Figure 3:**
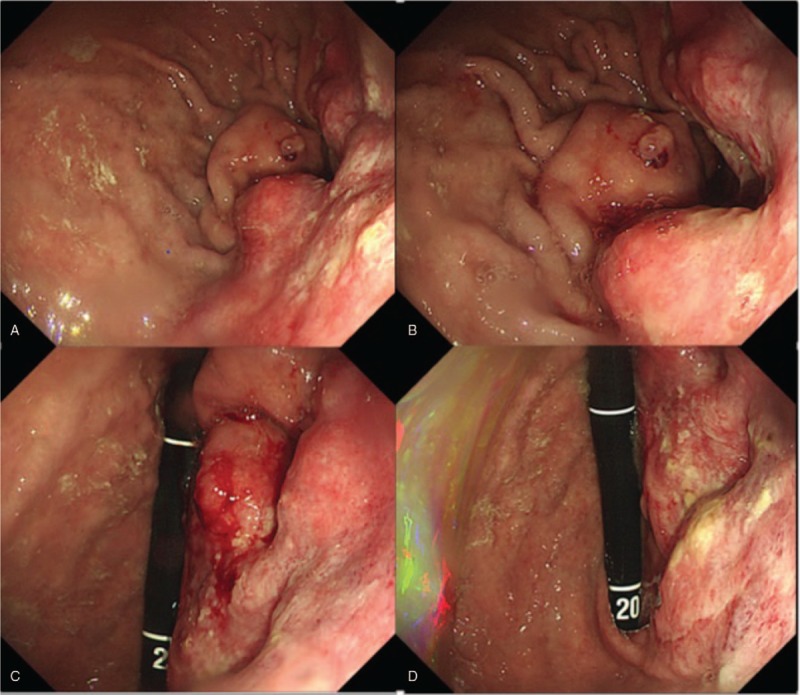
Esophagogastroduodenoscopy demonstrated previous subtotal gastrectomy with Billroth II anastomosis, but the afferent loop was not visible due to a fully obstructing mass at the anastomosis site of the remnant stomach.

For confirmation of the diagnosis and further management, esophagogastroduodenoscopy was performed. Endoscopy showed previous subtotal gastrectomy with Billroth II anastomosis, but the entrance of the afferent loop was not visible due to a fully obstructing mass at the anastomosis site of the remnant stomach (Fig. 3). The patient was suspected to have remnant gastric cancer, and biopsies were performed. Pathological examination of the endoscopic biopsy showed poorly differentiated adenocarcinoma with a signet ring cell component.

**Figure 4:**
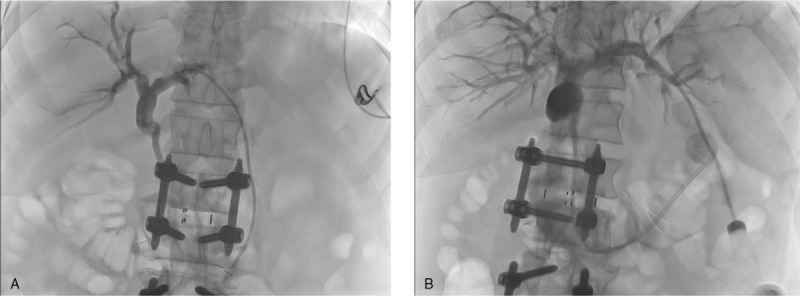
Cholangiography was performed via PTBD (percutaneous transhepatic biliary drainage) tube as an alternative for continuous opacification of the biliary tree to guide the insertion of a 10.2 French, multi side hole pig tail catheter via the left intrahepatic duct, with its tip being advanced into the afferent loop.

Three days later, cholangiography was performed via PTBD tube as an alternative for continuous opacification of the biliary tree, to guide the insertion of a 10.2 French, multiside hole pig tail catheter via the left intrahepatic duct, with its tip being advanced into the afferent loop (Fig. 4).

After PTBD reposition, he was able to tolerate a liquid diet without epigastric pain and discomfort. Amylase and lipase levels returned to the normal range. The patient's general condition and symptoms gradually improved during his hospitalization.

**Figure 5:**
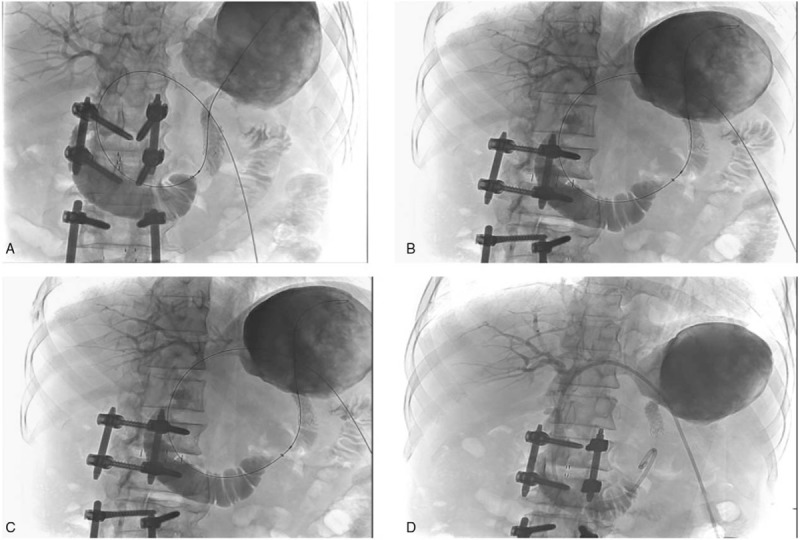
Stent placement. Jejunography confirmed the stricture of the afferent loop. A 12 mm × 80 mm self-expanding metal stent was placed across the stenosis via the transhepatic route.

Ten days after the PTBD reposition, a guide wire was inserted across the dilated loop and a catheter was then passed along the guide-wire to the gastric stricture caused by the tumor. A self-expanding metal stent (Taewoong Medical, Seoul, Korea), 12 mm in diameter and 80 mm in length, was successfully inserted through the stricture site (Fig. 5).

**Figure 6:**
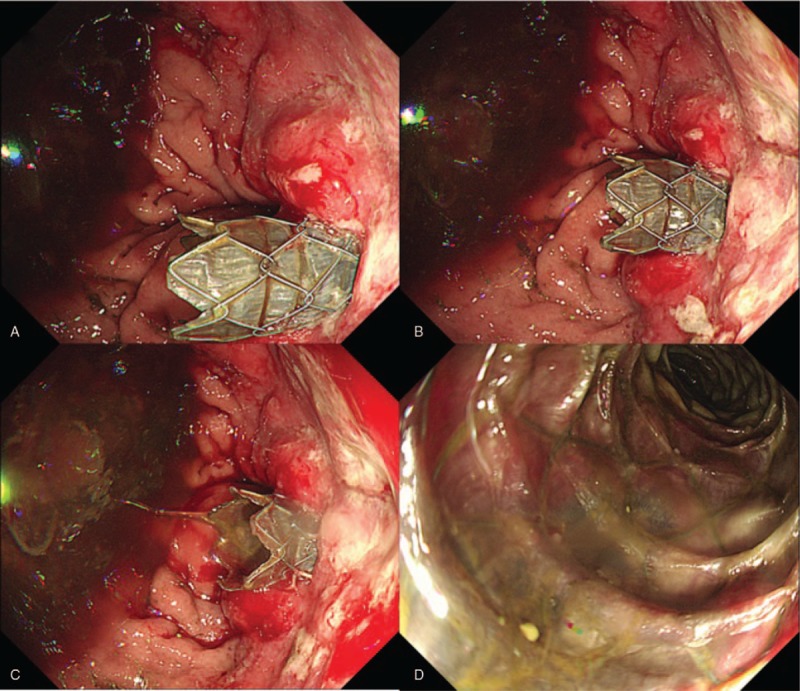
Follow-up esophagogastroduodenoscopy confirmed the presence of SEMS in the completely obstructed stenosis due to remnant gastric cancer (A–C). Endoscopy showed that bile was excreted in the afferent loop (D).

After stent insertion, follow-up esophagogastroduodenoscopy was performed. We confirmed the stent location at the stricture site that was previously completely obstructed due to remnant gastric cancer. The endoscopy scope was passed through the afferent loop and bile drainage was confirmed (Fig. 6).

After removing the PTBD tube, the patient was able to live comfortably without the need for a drainage catheter any longer. He died of disease progression 2 months later, without further recurrence of afferent loop syndrome.

## 3 Discussion

Our case demonstrates a rare complication with severe clinical manifestations of afferent loop syndrome. The advanced form of afferent loop syndrome due to remnant gastric cancer can lead to the development of a large volume enterobiliary reflux, resulting in ascending cholangitis and systemic sepsis due to bacterial overgrowth. Therapeutic strategies for afferent loop syndrome include surgical or nonsurgical treatments.^[5]^ Surgical procedures include radical resection and surgical bypass. Nonsurgical treatments can include drainage, balloon dilatation, and stent placement by the peroral, direct percutaneous, or percutaneous transhepatic approach. Traditionally, the only feasible treatment for correcting afferent loop syndrome was surgery. However, in 75% of patients with afferent loop syndrome, palliative surgical correction cannot be successfully performed because of poor general condition, peritoneal adhesions, or disseminated tumor.^[6,7]^

With recent advances in therapeutic endoscopy and interventional radiology, it has been possible to treat afferent loop syndrome with reduced rates of mortality and complications. In our case, the endoscopic approach to the afferent loop for balloon dilation or stenting may not have been possible because it was completely obscured. PTBD was a clinically effective treatment for decompression of an acutely dilated afferent loop, because the stricture of the afferent loop was easily accessible by the transhepatic route. Sufficient drainage of accumulated fluid in a dilated afferent loop is helpful for symptomatic relief via placement of the PTBD catheter tip in the duodenum. Subsequent placement of a self-expanding enteral stent achieved complete symptomatic resolution.

In case of severe cholangitis or biliary sepsis due to a dilated afferent loop, it is obvious that percutaneous drainage must be promptly performed. Subsequent stent placement along the tube should then be completed. In addition, when the bowel segment between the stomach and the lesion is too long or is tortuous, peroral stent placement can be difficult and a percutaneous approach should be considered. When choosing a treatment method, the patient's general condition, site of obstruction, postoperative anatomic variations, and associated symptoms must be taken into consideration. Delayed management of afferent loop obstruction may lead to severe complications, including perforation or necrosis of the afferent loop, resulting in death. Our case resulted from total occlusion of the stoma of the afferent loop and elevated intraluminal pressure secondary to remnant gastric cancer and a fully obstructing mass. The presentation of afferent loop syndrome due to remnant gastric cancer is an extremely rare event.

Previous studies showed that remnant gastric cancer is predominantly located on the anastomotic site due to the high risk of gastric mucosal damage secondary to duodenogastric reflux of bile carcinogens.^[3,8]^ This explains why Billroth II anastomosis is associated with a higher incidence of gastric cancer than Billroth I anastomosis. In our case, the patient had undergone subtotal gastrectomy with Billroth II anastomosis approximately 40 years prior. Hence, he had an increased risk of remnant gastric cancer. Although the incidence of gastric surgery for benign disease is decreasing, remnant gastric cancer secondary to benign disease occurs in the late postoperative period. Therefore, proper follow-up and observation after distal gastrectomy is very important to improve the survival rate of patients with remnant gastric cancer. Early diagnosis plays an important role in successfully performing curative resection and improving the prognosis for patients with remnant gastric cancer.

Our patient presented with factors that excluded the use of traditional surgical approaches. Owing to his poor general condition and the presence of ascites and the suspicion of peritoneal seeding, surgical treatment was not considered an appropriate therapeutic option. The treatment protocol described here might represent an appropriate conservative approach for patients who are poor surgical candidates. Our goal of extending the life of our patient was accomplished without significant postprocedural complications or recurrence of afferent loop syndrome. The patient lived approximately 2 months after the procedure.

## 4 Conclusion

In conclusion, we recommend that afferent loop syndrome should be considered in the differential diagnosis of patients with a history of subtotal gastrectomy with Billroth II anastomosis showing the typical radiographic presentation of dilated small bowel loop and clinical evidence of cholangitis. Early diagnosis, based on clinical symptoms and abdominal computed tomography findings, and prompt management are necessary to prevent life-threatening complications.

Our case shows that insertion of a metal stent via PTBD effectively treats ascending cholangitis and resolves afferent loop syndrome.

## Author contributions

**Conceptualization:** Ra Ri Cha, Wan Soo Kim, Jin Joo Kim, Jae Min Lee, Jin Kyu Cho.

**Data curation:** Su Beom Cho, Wan Soo Kim

**Investigation:** Jin Joo Kim, Jae Min Lee, Sang Soo Lee, Wan Soo Kim.

**Methodology:** Jin-Kyu Cho, Su Beom Cho.

**Resources:** Su Beom Cho, Wan Soo Kim

**Software:** Jin Joo Kim, Jae Min Lee, Sang Soo Lee

**Supervision:** Jin-Kyu Cho, Jin Joo Kim, Sang Soo Lee, Ra Ri Cha, Hyun Jin Kim.

**Writing – original draft:** Ra Ri Cha, Jin-Kyu Cho, Hyun Jin Kim.

**Writing – review & editing:** Ra Ri Cha, Sang Soo Lee, Hyun Jin Kim.

Ra Ri Cha orcid: 0000-0001-6024-9201.
